# Cryopreservation process alters the expression of genes involved in pathways associated with the fertility of bull spermatozoa

**DOI:** 10.3389/fgene.2022.1025004

**Published:** 2022-10-25

**Authors:** John Peter Ebenezer Samuel King, Manish Kumar Sinha, Arumugam Kumaresan, Pradeep Nag, Mohua Das Gupta, Mani Arul Prakash, Thirumala Rao Talluri, Tirtha Kumar Datta

**Affiliations:** ^1^ Theriogenology Laboratory, Veterinary Gynaecology and Obstetrics, Southern Regional Station of ICAR-National Dairy Research Institute, Bengaluru, Karnataka; ^2^ ICAR-Central Institute for Research on Buffaloes, Hisar, Haryana

**Keywords:** cryopreservation, spermatozoa, pathways, transcripts, fertility, bovine

## Abstract

In bovines, cryopreserved semen is used for artificial insemination; however, the fertility of cryopreserved semen is far lower than that of fresh semen. Although cryopreservation alters sperm phenotypic characteristics, its effect on sperm molecular health is not thoroughly understood. The present study applied next-generation sequencing to investigate the effect of cryopreservation on the sperm transcriptomic composition of bull spermatozoa. While freshly ejaculated bull spermatozoa showed 14,280 transcripts, cryopreserved spermatozoa showed only 12,375 transcripts. Comparative analysis revealed that 241 genes were upregulated, 662 genes were downregulated, and 215 genes showed neutral expression in cryopreserved spermatozoa compared to fresh spermatozoa. Gene ontology analysis indicated that the dysregulated transcripts were involved in nucleic acid binding, transcription-specific activity, and protein kinase binding involving protein autophosphorylation, ventricular septum morphogenesis, and organ development. Moreover, the dysregulated genes in cryopreserved spermatozoa were involved in pathways associated with glycogen metabolism, MAPK signalling, embryonic organ morphogenesis, ectodermal placode formation, and regulation of protein auto-phosphorylation. These findings suggest that the cryopreservation process induced alterations in the abundance of sperm transcripts related to potential fertility-associated functions and pathways, which might partly explain the reduced fertility observed with cryopreserved bull spermatozoa.

## Introduction

Artificial insemination using cryopreserved semen is widely and routinely practiced for the genetic improvement of livestock. Ultralow freezing allows the preservation of semen for several years, which can be used later for artificial breeding (Barbas and Mascarenhas, 2009). Although the cryopreservation of sperm is an important process in assisted reproduction technologies, the fertility of cryopreserved spermatozoa is reportedly not as good as fresh spermatozoa (Watson, 1995; Bailey et al., 2000; Kadirvel et al., 2009). Several reasons are attributed to the decreased fertility of cryopreserved semen, primarily damage that occurs in spermatozoa during cryopreservation (cryodamage), which alters its fertilising potential (Cormier et al., 1997; Watson, 2000; Yeste, 2016). The process of cryopreservation results in the death of almost 50% of spermatozoa, while the remaining sperm population shows altered functional competencies (Kumaresan et al., 2011; Kumaresan et al., 2012; Singh et al., 2016; Kumaresan et al., 2017; Saraf et al., 2019; Rather et al., 2020; Vignesh et al., 2020; Nag et al., 2021), which might be linked to the reduced fertility of cryopreserved semen. However, the fertility of cryopreserved semen subjected to sperm selection methods (for the selection of viable, active, and phenotypically normal spermatozoa) was also not as good as that of fresh semen, although some improvement in fertility was observed (Said and Land, 2011; Marzano et al., 2020). Therefore, besides cryopreservation-induced sperm structural and functional alterations, other inherent factors in spermatozoa might also be altered during cryopreservation as cryopreserved spermatozoa with normal phenotypic characteristics also show altered fertilising potential compared to fresh spermatozoa (Kadirvel et al., 2009; Elango et al., 2021). Therefore, the assessment of the molecular alterations induced by the process of cryopreservation is essential for understanding the decreased fertility associated with cryopreserved semen.

Sperm was previously believed to deliver only the paternal DNA to the oocyte after fertilisation. However, several recent studies have demonstrated the role of sperm transcripts in male fertility (Paul et al., 2020; Prakash et al., 2021; Saraf et al., 2021) and subsequent embryonic development (Zhang et al., 2018). After fertilisation, spermatozoal mRNAs are transferred to the oocyte and play important roles in embryonic development, morphogenesis, and implantation (Ostermeier et al., 2004). Bull spermatozoa harbours a repertoire of transcripts (Prakash et al., 2021) that vary with semen quality and fertility (Karuthadurai et al., 2022). Earlier studies reported different transcriptomic profiles between epididymal and ejaculated spermatozoa, and also among different seasons (AL-Sahaf, 2012), indicating the influence of the micro-environment on sperm transcripts. Any changes in the transcript content of the sperm affect sperm properties such as motility, DNA intactness, and acrosome integrity (Bissonnette et al., 2009).

Although structural damage to sperm during cryopreservation has been studied extensively, the molecular alterations induced by the process of cryopreservation are not well documented. We hypothesised that the transcriptomic composition of sperm could be influenced by the process of cryopreservation, which might be associated with the reduced fertility of cryopreserved semen. Therefore, in the present study, we assessed the global transcriptomic composition of spermatozoa immediately after ejaculation and after cryopreservation using next-generation sequencing. We compared the transcriptomic profile of fresh spermatozoa to that of cryopreserved spermatozoa and identified common and dysregulated transcripts. Using functional annotation of these genes, we report the alterations in sperm transcripts and important pathways associated with fertility, which were induced by the cryopreservation process.

## Materials and methods

### Ethical approval statement

The current study was carried out at the Theriogenology Laboratory of the Southern Regional Station of ICAR-National Dairy Research Institute, Bengaluru, Karnataka. All the experiments and procedures performed in this study were approved by the Animal Ethical Committee of the institute (CPCSEA/IAEC/LA/SRS-ICAR-NDRI-2019/No.04).

### Experimental bulls and sample preparation

This investigation was conducted on Holstein-Friesian crossbred bulls (*n* = 6; age 4–6 years). All experimental bulls had passed breeding soundness evaluations and were routinely used for artificial breeding. Ejaculates were collected using an artificial vagina; after preliminary evaluation, only ejaculates with minimum sperm concentrations of 600 million/ml, +3.0 mass activity (0–5 scale), ≥70% progressive individual motility, and <20% sperm abnormalities were utilised for further processing. The ejaculates were divided into two aliquots; one aliquot was used fresh while the other aliquot was subjected to cryopreservation as per the standard procedure. Briefly, the ejaculates were diluted using pre-warmed (34°C) Tris-egg yolk glycerol extender (20% egg yolk and 7% glycerol fractions) and then further processed for cryopreservation. The diluted semen was then filled and sealed in 0.25 ml mini straws (20 × 10^6^ sperm per dose) using an automatic filling and sealing machine. The straws were then equilibrated in a cold handling cabinet (IMV Technologies, France) for 4 h at 4°C. Post-equilibration, the doses were loaded into a programmable Biofreezer (Digitcool, IMV Technologies, France) for cryopreservation as per standard protocol. After reaching −140°C, the straws were directly plunged into liquid nitrogen. After cryopreservation, the frozen semen was thawed at 37°C for 30 s and used for further processing. Ejaculates from three bulls were pooled to obtain one representative sample. Therefore, we obtained two representative samples from six bulls for each condition (fresh and cryopreserved), which were individually subjected to transcriptomic analysis.

### RNA extraction and synthesis of cDNA

Discontinuous Percoll gradients (90–45%) were used to fractionate pure sperm by eliminating epithelial cells and seminal plasma. The method described by Parthipan et al. (2015), with minor modifications was used for the extraction of total sperm RNA from fresh and cryopreserved samples using TRIzol (Ambion, Thermo Fisher Scientific, United States). The RNA was quantified using Nanodrop (ND-1000, Thermo-scientific, United States). For cDNA library preparation, RNA samples with 260/280 ratios of 1.85–2.0 were used. An initial concentration of 50–100 ng of RNA was used for cDNA synthesis using the RevertAid First Strand cDNA Synthesis Kit (Thermo Fisher Scientific, United States). The synthesised cDNA was stored at −20°C until further use.

### Transcriptomics library preparation

Total RNA (1 μg) enriched for mRNA using the NEB Magnetic mRNA Isolation Kit (Illumina, United States). RNA library preparation was performed using the NEB Ultra II RNA library prep kit (Illumina, United States) and sequencing (paired-end technology) was performed on an Illumina NextSeq 500 instrument (Illumina, United States. The enriched mRNA was fragmented to 200 bp using fragmentation buffer. Complementary RNA sequence hybridisation was performed by adding random hexamer primers. Reverse transcriptase enzyme and dNTPs were used for synthesising the first strand of cDNA from fragments. DNA polymerase I and RNase H were used to convert the single-strand cDNA into double-stranded cDNA, which was purified using 1.8x AMPure beads. Adaptor-ligated cDNA was purified using AMPure beads and was enriched with specific primers for sequencing on the Illumina platforms.

### RNA sequencing and data analysis

The Galaxy online server tool was used to analyse the sequences. The quality of the raw generated data was checked using the Fast QC program with a Phred quality score cut-off of Q30. The Cutadapt tool was used to remove adaptor sequences from the FASTQ files. HISAT2 (version2.1) was used to align the sequences to the reference genome. The samples were aligned to the reference genome of *Bos taurus* (version UCD 3.1.94). Cufflinks (version—2.2.1.2) was used to identify and estimate the abundance of the transcripts. After normalisation, the transcript expression levels were calculated as FPKM (fragments per kilobase of transcript per million mapped reads). Full-length transcript analysis was performed using the depth of coverage program version-0.0.2 in GATK.

### Functional annotation and Gene Ontology analysis

Functional annotation and gene ontology (GO) analysis were performed using the Database for Annotation, Visualization, and Integrated Discovery (DAVID) Bioinformatics Resources (v6.8) (https://david.ncifcrf.gov/). Molecular Function (MF), Biological Process (BP), cellular components (CC), and Kyoto Encyclopedia of Genes and Genomes (KEGG) pathway analyses were performed to identify the genes with variations. For network analysis, genes related to sperm quality were selected from MF, BP, CC, and KEGG. ClueGo (Version 2.5.4) plugins in the open-source Cytoscape software (version 3.7.1) (Cluego.org) were used to identify the interactions of novel genes and their associated pathways.

### Statistical analysis

DAVID was used to perform functional enrichment analysis of the Gene Ontology categories and KEGG pathway elements. The EASE score was calculated using the Fisher’s exact *p*-value based on the input list of genes and genes participating in a certain pathway, resulting in a strongly enriched word. A *p*-value threshold of 0.01 was used to determine the EASE score cut-off. After enrichment analysis, terms with *p*-values <0.01 were identified as enriched terms. Thus, the lower the *p*-value, the more enriched the phrase. At least two genes were required for the examination of connection. The corrected *p*-values obtained after adjustments using the Benjamini and Bonferroni tests were defined as the false discovery rate (FDR). The processed reads were mapped to the reference genome for analysis of the raw transcriptomic data. The number of reads mapped to the exonic region indicated the gene expression. The read counts were used by DESeq to determine the differential expression based on the number of mapped reads. DESeq was used to create size factors (using the size factors function) and fit the data with a negative binomial distribution (using the nbinom test function) to compare the control (fresh) and treated (cryopreserved) groups and return log2-fold changes and significant *p*-values.

## Results

### Differentially regulated transcripts

**FIGURE 1:**
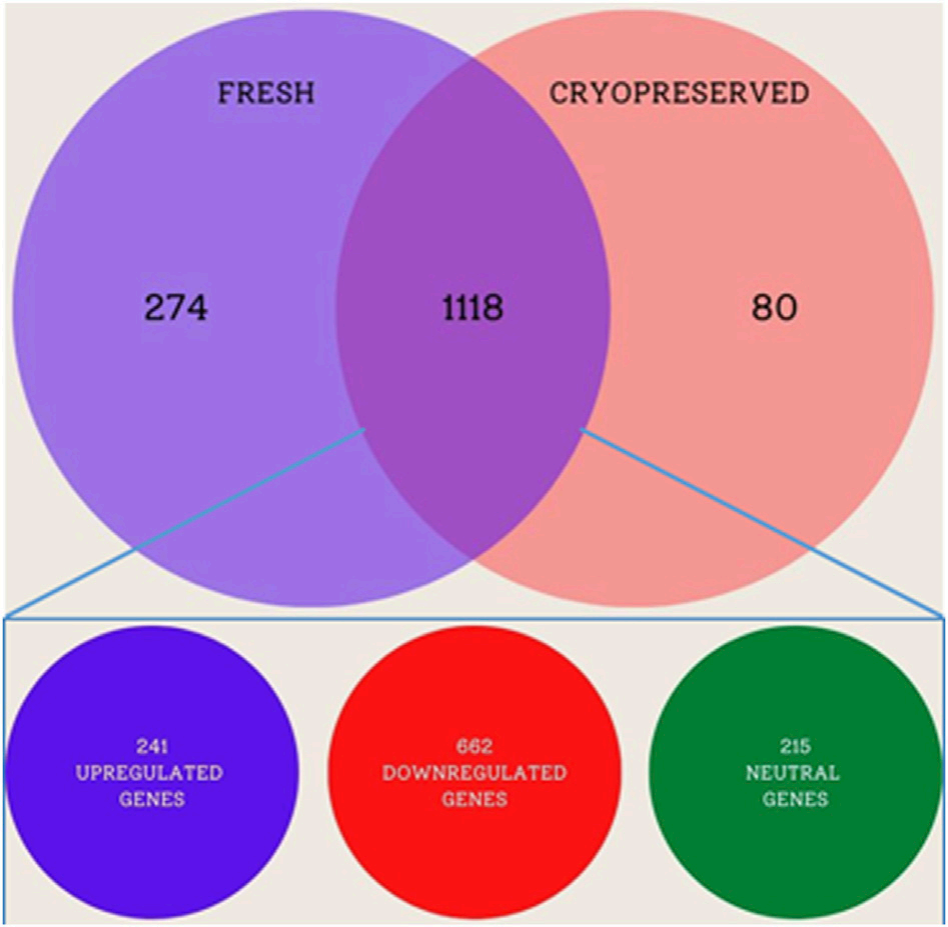
Venn diagram of the transcriptomic compositions of fresh and cryopreserved bull spermatozoa.

**FIGURE 2:**
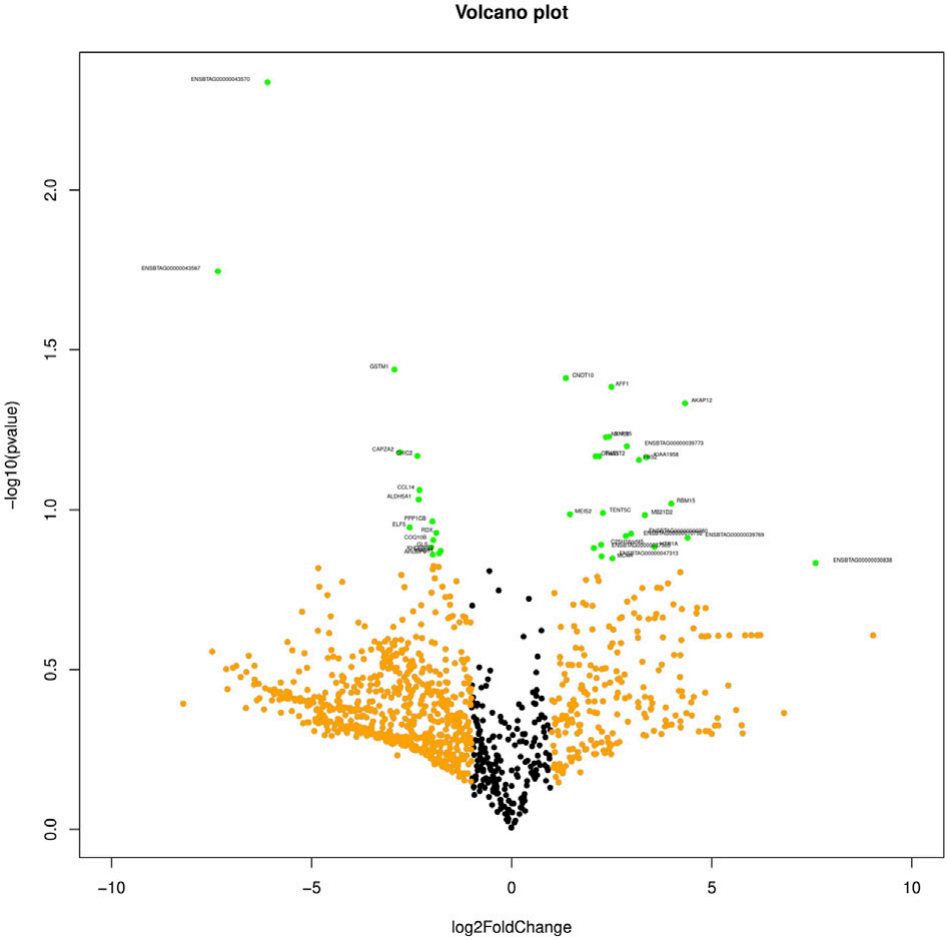
Volcano plot representing the gene log-fold changes and *p*-value. Green dots: significantly differentially expressed genes.

**FIGURE 3:**
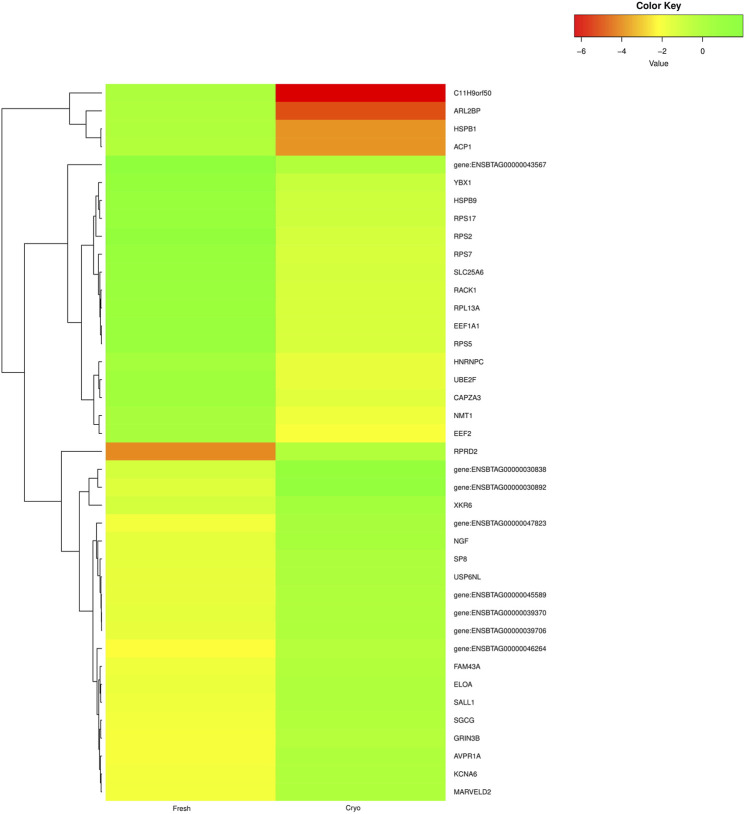
Heat map representing the top 20 upregulated (green) and top 20 downregulated (red and yellow) genes in cryopreserved spermatozoa compared to fresh spermatozoa.

The primary analysis of fresh and cryopreserved spermatozoa resulted in 14,280 and 12,375 transcripts, respectively (Supplementary File S1 and Supplementary Figure S1) indicating that the process of cryopreservation altered the sperm transcriptomic profile. After normalisation of the read counts, a total of 1118 genes were common between fresh and cryopreserved spermatozoa. In cryopreserved spermatozoa, 241 genes were upregulated, 662 genes were downregulated, and 215 genes were neutral (neither up- regulated nor downregulated) in expression compared to fresh spermatozoa (Figure 1). The genes that were upregulated or downregulated based on log2 fold-change values are shown in a volcano plot (Figure 2). A heat map of the top 20 upregulated and top 20 downregulated genes in the cryopreserved sample (Figure 3) showed the genes with highly dysregulated expression. *C11H9orf50*, *ARL2BP*, and *HSPB1* were the most downregulated, while *RPRD2*, *ENSBTAG00000030892*, and *ENSBTAG00000030838* were the most upregulated in cryopreserved spermatozoa.

### Gene Ontology

**FIGURE 4:**
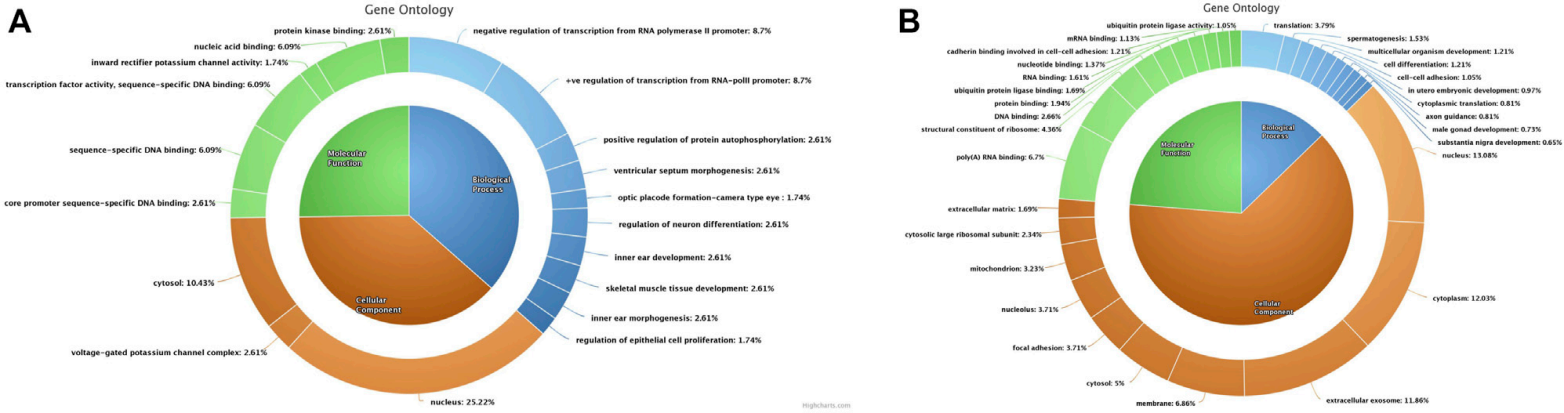
Gene Ontology chart of differentially expressed genes between fresh and cryopreserved spermatozoa. All three components (molecular function, biological process, and cellular functions) are represented in the donut chart. **(A)** Upregulated genes. **(B)** Downregulated genes.

The dysregulated genes were selected for Gene Ontology (GO) analysis based on a log2 fold-change cut-off of ±1. These dysregulated genes were then subjected to DAVID analysis to annotate GO terms including molecular function (MF), biological process (BP), and cellular components (CC). A total of 54 upregulated and 411 downregulated genes were annotated for GO analysis. From these, the top MF, BP, and CC for upregulated and downregulated genes (Figures 4A,B) (Supplementary File S2) were selected based on the counts of genes involved in each GO term. The important upregulated genes in the cryopreserved spermatozoa were involved in specific DNA binding, nucleic acid binding, transcription-specific activity, protein kinase binding, core promotor sequence-specific DNA binding, and inward rectifier potassium channel activity. These were mostly localised in the nucleus, cytosol, and potassium channel complex and were involved in biological processes such as the regulation of transcription from the RNA polymerase II promotor, the regulation of neuronal differentiation, the positive regulation of protein autophosphorylation, ventricular septum morphogenesis, organ development, Rap protein signal transduction, neuroblast proliferation, and negative regulation of myeloid cell differentiation. The downregulated genes were involved in poly(A) RNA binding, structural constituents of ribosomes, DNA binding, protein binding, RNA binding, ubiquitin protein ligase binding, and nucleotide binding.

### KEGG pathway and network analysis

**FIGURE 5:**
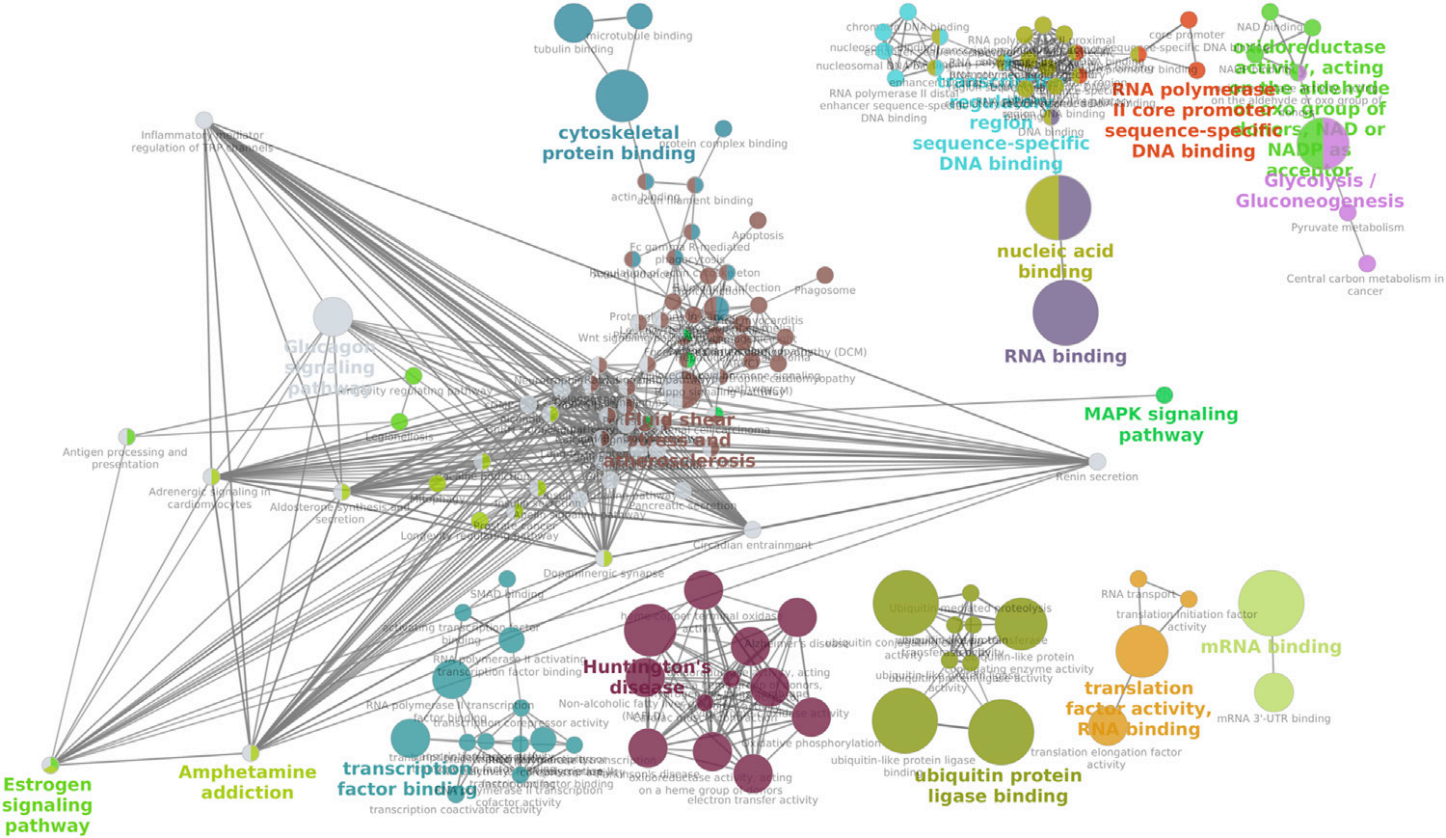
Network analysis of all downregulated genes in the cryopreserved samples. MAPK signalling pathway, fluid shear and stress atherosclerosis, cytoskeletal protein binding, and the oestrogen signalling pathway are mapped.

**FIGURE 6:**
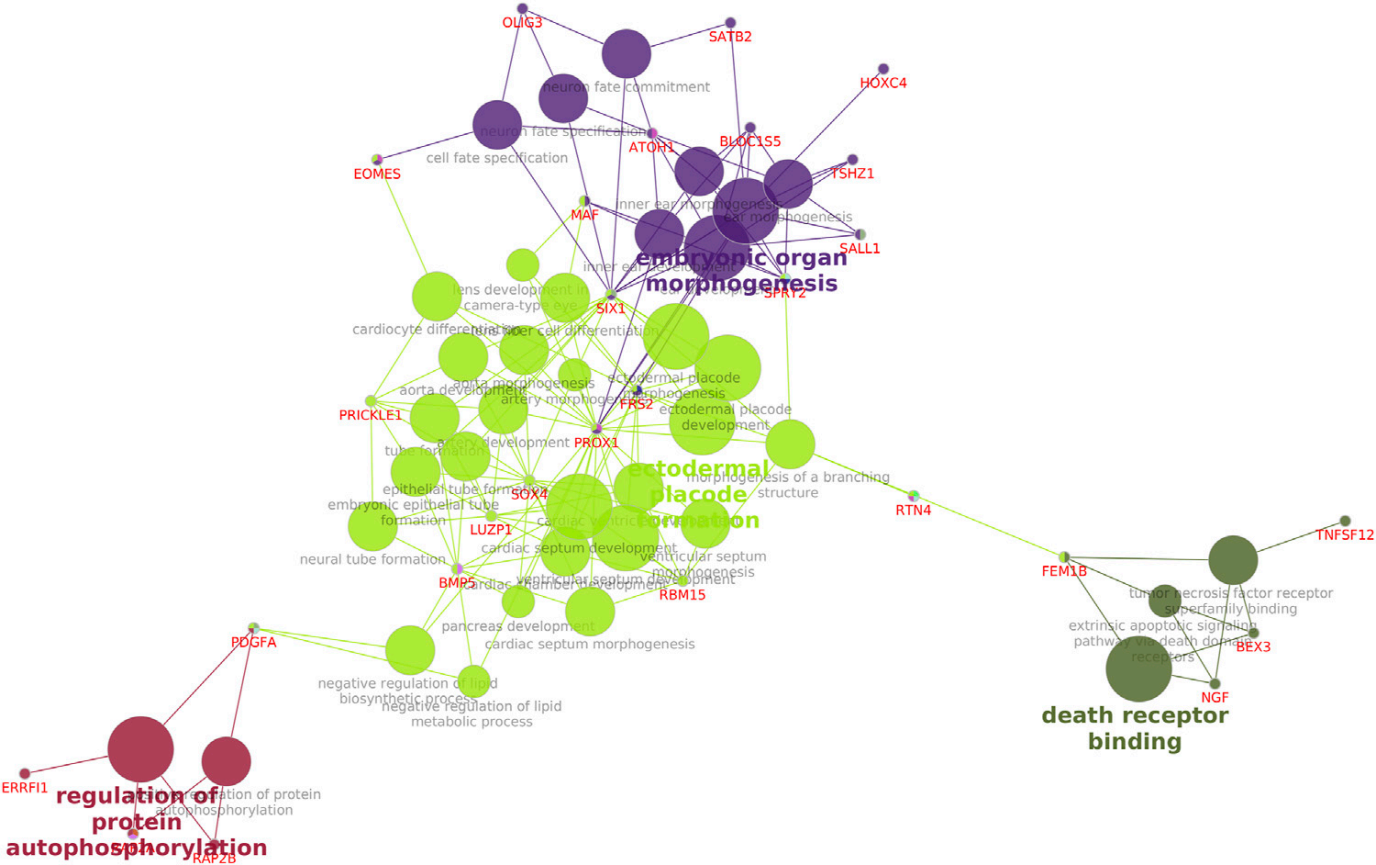
Network analysis of all upregulated genes in the cryopreserved samples. Embryonic organ morphogenesis, ectodermal placode formation, regulation of protein auto-phosphorylation, and death receptor binding are mapped.

KEGG pathway enrichment analysis of the downregulated genes (Supplementary File S3) in cryopreserved spermatozoa revealed the involvement of genes in important pathways related to ribosomes (50 genes), Huntington’s disease (17 genes), thermogenesis (16 genes), spliceosomes (16 genes), and endocytosis (16 genes). KEGG pathway enrichment analysis of the upregulated genes (Supplementary File S3) in cryopreserved spermatozoa revealed the involvement of genes in pathways related to the neurotrophin signalling pathway (4 genes), transcriptional misregulation in cancer (4 genes), and neuroactive ligand-receptor interaction (5 genes). A network analysis of GO terms and KEGG pathways for upregulated and downregulated genes related to fertility was also performed. The network analysis of downregulated (Figure 5) genes in cryopreserved spermatozoa showed their involvement in pathways such as glycogen metabolism, the MAPK signalling pathway, fluid shear and stress atherosclerosis, cytoskeletal protein binding, the oestrogen signalling pathway, amphetamine addiction, transcription factor binding, Huntington’s disease, ubiquitin protein ligase binding, translation factor activity RNA binding, mRNA binding, transcription regulation region sequence-specific DNA binding, RNA polymerase II core promoter sequence-specific DNA binding, oxidoreductase activity, and glycolysis/gluconeogenesis. The network analysis of upregulated (Figure 6) genes in cryopreserved spermatozoa showed their involvement in pathways such as embryonic organ morphogenesis, ectodermal placode formation, regulation of protein auto-phosphorylation, and death receptor binding (Supplementary File S4).

## Discussion

The process of cryopreservation alters sperm phenome and functions, which affect the quality and fertility of spermatozoa. Recent studies have shown that sperm transcripts are associated with sperm quality and fertility, as well as embryonic development (Zhang et al., 2018; Paul et al., 2020; Prakash et al., 2021). Since the process of cryopreservation alters sperm structure, this process may also affect the transcriptomic composition of sperm. The results of the present study demonstrated the significant alterations in the transcriptomic composition of bull sperm by the process of cryopreservation, which might explain the reduced fertility of cryopreserved semen.

The mature spermatozoal RNA undergoes certain cellular modifications during spermiogenesis. The required mRNAs are then packed in spermatozoa before it reaches the transcriptionally nascent stage in the epididymis (Grunewald et al., 2005; Vijayalakshmy et al., 2018; Paul et al., 2020) for delivery to the oocyte after fertilisation. We observed highly downregulated expression of *C11H9orf50*, *ARL2BP*, and *HSPB1* and highly upregulated expression of *RPRD2*, *ENSBTAG00000030892*, and *ENSBTAG00000030838* in cryopreserved spermatozoa. *ARL2BP* is required for ciliary microtubule structure. A mouse *ARL2BP* knockout showed structural impairments such as abnormal head and misassembled tail of sperm (Moye et al., 2019). *HSBP1* is responsible for organising the muscle cytoskeleton. *HSBP1* (also known as *HSP27*) is important for muscle formation. A mouse *HSBP1* knockout showed destructured myofibrils and increased gaps between myofibrils (Kammoun et al., 2016). *RPRD2* is an RNA polymerase II interacting protein that co-purifies with RPRD1A, RPRD1B, and RPRD2 and contains serine and proline-rich regions (Ni et al., 2011). The proline-rich domains function as docking sites for signalling protein modules and play important roles in protein-protein interactions (Chin et al., 1997; Elias et al., 2020). The overexpression of this gene might alter transcription, thus hampering protein interactions.

The results of the network analysis showed downregulation of cGMP/PKG signalling, which is responsible for sperm capacitation through Ca^2+^ and tyrosine phosphorylation in the presence of C-type natriuretic peptide (CNP) (Wu et al., 2019). CNP, which is localised in the acrosomal region of the sperm head and tail, has a dose-dependent role in acrosome reaction and motility (Xia et al., 2016). CNP might be affected by the process of cryopreservation, as fast freezing increases the ice-liquid interface with protein molecules, thereby increasing protein damage (Cao et al., 2003), which in turn affects sperm motility and acrosome reaction. The MAPK signalling pathway is involved in sperm development and function through spermatogenesis and fertilising potential. In mature spermatozoa, the MAPK signalling pathway plays an important role in acrosome reaction, hyperactivation, and motility (Almog and Naor, 2010; Ebenezer Samuel King et al., 2022). We also observed the downregulation of cytoskeletal protein binding pathways in the cryopreserved sample. These pathways are linked to tubulin binding. Actin, along with the cytoskeletal protein tubulin, is involved in the regulation of sperm motility, capacitation, and acrosome reaction (Salvolini et al., 2013). In normal spermatogenesis, oestrogen signalling through ESR1 is essential. Human spermatozoa contain functional aromatase, which is expressed even after ejaculation. Aromatase, in the presence of oestrogen receptor, has functions including sperm mobility and fertilising ability (Carreau et al., 2011; Cooke and Walker, 2022). The ubiquitin ligase complex, which is required for caspase regulation, was also downregulated in cryopreserved spermatozoa. Caspase is an important protease required for apoptosis. The activation of caspase during sperm differentiation is regulated by the ubiquitin ligase complex (Arama et al., 2007). mRNA binding proteins are needed for the differentiation of spermatids into spermatozoa by modulating the expression of specific mRNAs (Wishart and Dixon, 2002). All the pathways important for spermatozoa function, especially motility, capacitation, and acrosome reaction, were downregulated in the cryopreserved spermatozoa in the present study.

Genes involved in the embryo organ morphogenesis pathway were upregulated in cryopreserved spermatozoa. This pathway is required for tissue and organ development to perform special functions during the embryonic stage. Among these, *SPRY2* is required for the normal development of genitalia; *TSHZ1* is required for the development of the soft palate, middle ear, and axial skeleton; and *SATB2* codes for the jaw development (Britanova et al., 2006; Coré et al., 2007; Ching et al., 2014). The overexpression of these genes might lead to abnormal embryo development. *PRICKLE 1* is involved in ectodermal placode formation and also plays a role in nervous system development and nerve cell movement. A polymorphism in *PRICKLE 1* reportedly affected acrosome integrity and DNA quality in cryopreserved spermatozoa (Mańkowska et al., 2020). The regulation of protein autophosphorylation, one of the upregulated pathways in cryopreserved spermatozoa, is involved in the positive regulation of protein autophosphorylation. *RAP2B* in protein autophosphorylation codes for miR-205; miR-205 overexpression inhibits the PI3K/AKT signalling pathway (Cui et al., 2020). Earlier studies also showed that cryopreservation suppressed the expression of important pathways and genes in sperm, causing DNA fragmentation and morphological deformation (Hossen et al., 2021). Moreover, sperm stored in liquid nitrogen for longer times showed significantly increased abnormalities (Malik et al., 2015). A study in zebrafish showed that cryopreservation was the major cause of genetic and epigenetic changes in germ cells (Riesco and Robles, 2013). Our findings demonstrated the alteration of the transcriptional abundances of genes involved in important pathways in sperm function and fertility by the process of cryopreservation. These findings open new avenues for targeted studies on individual pathways and the development of ameliorative measures to minimise the effect of cryopreservation on the molecular health of sperm.

## Conclusion

In conclusion, the transcriptional abundances of many genes were altered in cryopreserved spermatozoa, leading to changes in pathways important for sperm function and fertility. Earlier studies also indicated that the cryopreservation process altered the sperm expression levels of genes crucial for fertilisation and early embryo development (Valcarce et al., 2013), consistent with our observations. The cryopreservation process induced alterations in the abundance of sperm transcripts and potential fertility-associated pathways, including glycogen metabolism, cGMP/PKG signalling, MAPK signalling, and the ubiquitin ligase complex, which could be a possible explanation for the reduced fertility observed in cryopreserved bull spermatozoa.

## Data Availability

The datasets presented in this study can be found in online repositories. The names of the repository/repositories and accession number(s) are as shown below: https://www.ncbi.nlm.nih.gov/, PRJNA847399; https://www.ncbi.nlm.nih.gov/, PRJNA516089.

## References

[B1] Al-Sahaf M. M. (2012). Monthly changes in testes and epididymis measurements with some semen characteristics of tail epididymis for Iraqi buffalo: Mohammed Mehdi AL-Sahaf and Najlaa Sami Ibrahim. Iraqi J. Vet. Med. 36, 204–208. 10.30539/iraqijvm.v36i2.497

[B2] Almog T. Naor Z. (2010). The role of Mitogen activated protein kinase (MAPK) in sperm functions. Mol. Cell. Endocrinol. 314, 239–243. 10.1016/j.mce.2009.05.009 19467295

[B3] Arama E. Bader M. Rieckhof G. E. Steller H. (2007). A ubiquitin ligase complex regulates caspase activation during sperm differentiation in Drosophila. PLoS Biol. 5, e251. 10.1371/journal.pbio.0050251 17880263 PMC1976628

[B4] Bailey J. L. Bilodeau J. F. Cormier N. (2000). Semen cryopreservation in domestic animals: A damaging and capacitating phenomenon. J. Androl. 21, 1–7. 10670514

[B5] Barbas J. P. Mascarenhas R. D. (2009). Cryopreservation of domestic animal sperm cells. Cell Tissue Bank. 10, 49–62. 10.1007/s10561-008-9081-4 18548333

[B6] Bissonnette N. Lévesque-Sergerie J-P. Thibault C. Boissonneault G. (2009). Spermatozoal transcriptome profiling for bull sperm motility: A potential tool to evaluate semen quality. Reproduction 138, 65–80. 10.1530/REP-08-0503 19423662

[B7] Britanova O. Depew M. J. Schwark M. Thomas B. L. Miletich I. Sharpe P. (2006). Satb2 haploinsufficiency phenocopies 2q32-q33 deletions, whereas loss suggests a fundamental role in the coordination of jaw development. Am. J. Hum. Genet. 79, 668–678. 10.1086/508214 16960803 PMC1592575

[B8] Cao E. Chen Y. Cui Z. Foster P. R. (2003). Effect of freezing and thawing rates on denaturation of proteins in aqueous solutions. Biotechnol. Bioeng. 82, 684–690. 10.1002/bit.10612 12673768

[B9] Carreau S. Bois C. Zanatta L. Silva F. R. M. B. Bouraima-Lelong H. Delalande C. (2011). Estrogen signaling in testicular cells. Life Sci. 89, 584–587. 10.1016/j.lfs.2011.06.004 21703280

[B10] Chin K-C. Li G. G-X. Ting J. P-Y. (1997). Importance of acidic, proline/serine/threonine-rich, and GTP binding regions in the major histocompatibility complex class II transactivator: Generation of transdominant-negative mutants. Proc. Natl. Acad. Sci. U. S. A. 94, 2501–2506. 10.1073/pnas.94.6.2501 9122224 PMC20117

[B11] Ching S. T. Cunha G. R. Baskin L. S. Basson M. A. Klein O. D. (2014). Coordinated activity of Spry1 and Spry2 is required for normal development of the external genitalia. Dev. Biol. 386, 1–11. 10.1016/j.ydbio.2013.12.014 24361260 PMC3921686

[B12] Cooke P. S. Walker W. H. (2022). Nonclassical androgen and estrogen signaling is essential for normal spermatogenesis. Semin. Cell Dev. Biol. 121, 71–81. 10.1016/j.semcdb.2021.05.032 34119408

[B13] Coré N. Caubit X. Metchat A. Boned A. Djabali M. Fasano L. (2007). Tshz1 is required for axial skeleton, soft palate and middle ear development in mice. Dev. Biol. 308, 407–420. 10.1016/j.ydbio.2007.05.038 17586487

[B14] Cormier N. Sirard M. A. Bailey J. L. (1997). Premature capacitation of bovine spermatozoa is initiated by cryopreservation. J. Androl. 18, 461–468. 9283961

[B15] Cui Y. Chen R. Ma L. Yang W. Chen M. Zhang Y. (2020). miR-205 expression elevated with EDS treatment and induced leydig cell apoptosis by targeting RAP2B via the PI3K/AKT signaling pathway. Front. Cell Dev. Biol. 8, 448. 10.3389/fcell.2020.00448 32596241 PMC7300349

[B16] Ebenezer Samuel King J. P. Kumaresan A. Talluri T. R. Sinha M. K. Raval K. Nag P. (2022). Genom-wide analysis identifies single nucleotide polymorphism variations and altered pathways associated with poor semen quality in breeding bulls. Reprod. Domest. Anim. 10.1111/rda.14185 35702937

[B17] Elango K. Kumaresan A. Ashokan M. Karuthadurai T. Nag P. Bhaskar M. (2021). Dynamics of mitochondrial membrane potential and DNA damage during cryopreservation of cattle and buffalo bull spermatozoa. Indian J. Anim. Sci. 91, 9–14.

[B18] Elias R. D. Ma W. Ghirlando R. Schwieters C. D. Reddy V. S. Deshmukh L. (2020). Proline-rich domain of human ALIX contains multiple TSG101-UEV interaction sites and forms phosphorylation-mediated reversible amyloids. Proc. Natl. Acad. Sci. U. S. A. 117, 24274–24284. 10.1073/pnas.2010635117 32917811 PMC7533887

[B19] Grunewald S. Paasch U. Glander H-J. Anderegg U. (2005). Mature human spermatozoa do not transcribe novel RNA. Andrologia 37, 69–71. 10.1111/j.1439-0272.2005.00656.x 16026427

[B20] Hossen S. Sukhan Z. P. Cho Y. Kho K. H. (2021). Effects of cryopreservation on gene expression and post thaw sperm quality of pacific abalone, Haliotis discus hannai. Front. Mar. Sci. 8, 652390. 10.3389/fmars.2021.652390

[B21] Kadirvel G. Kumar S. Kumaresan A. Kathiravan P. (2009). Capacitation status of fresh and frozen-thawed buffalo spermatozoa in relation to cholesterol level, membrane fluidity and intracellular calcium. Anim. Reprod. Sci. 116, 244–253. 10.1016/j.anireprosci.2009.02.003 19261396

[B22] Kammoun M. Picard B. Astruc T. Gagaoua M. Aubert D. Bonnet M. (2016). The invalidation of HspB1 gene in mouse alters the ultrastructural phenotype of muscles. PLoS ONE 11, e0158644. 10.1371/journal.pone.0158644 27512988 PMC4981447

[B23] Karuthadurai T. Das D. N. Kumaresan A. Sinha M. K. Kamaraj E. Nag P. (2022). Sperm transcripts associated with odorant binding and olfactory transduction pathways are altered in breeding bulls producing poor-quality semen. Front. Vet. Sci. 9, 799386. 10.3389/fvets.2022.799386 35274020 PMC8902071

[B24] Kumaresan A. Johannisson A. Al-Essawe E. M. Morrell J. M. (2017). Sperm viability, reactive oxygen species, and DNA fragmentation index combined can discriminate between above- and below-average fertility bulls. J. Dairy Sci. 100, 5824–5836. 10.3168/jds.2016-12484 28478003

[B25] Kumaresan A. Johannisson A. Humblot P. Bergqvist A-S. (2012). Oviductal fluid modulates the dynamics of tyrosine phosphorylation in cryopreserved boar spermatozoa during capacitation. Mol. Reprod. Dev. 79, 525–540. 10.1002/mrd.22058 22674908

[B26] Kumaresan A. Siqueira A. P. Hossain M. S. Bergqvist A. S. (2011). Cryopreservation-induced alterations in protein tyrosine phosphorylation of spermatozoa from different portions of the boar ejaculate. Cryobiology 63, 137–144. 10.1016/j.cryobiol.2011.08.002 21893053

[B27] Malik A. Laily M. Zakir M. I. (2015). Effects of long term storage of semen in liquid nitrogen on the viability, motility and abnormality of frozen thawed Frisian Holstein bull spermatozoa. Asian Pac. J. Reproduction 4, 22–25. 10.1016/S2305-0500(14)60052-X

[B28] Mańkowska A. Brym P. Paukszto L. Jastrzębski J. P. Fraser L. (2020). Gene polymorphisms in boar spermatozoa and their associations with post-thaw semen quality. Int. J. Mol. Sci. 21, 1902. 10.3390/ijms21051902 PMC7084667 32164368

[B29] Marzano G. Chiriacò M. S. Primiceri E. Dell’Aquila M. E. Ramalho-Santos J. Zara V. (2020). Sperm selection in assisted reproduction: A review of established methods and cutting-edge possibilities. Biotechnol. Adv. 40, 107498. 10.1016/j.biotechadv.2019.107498 31836499

[B30] Moye A. R. Bedoni N. Cunningham J. G. Sanzhaeva U. Tucker E. S. Mathers P. (2019). Mutations in ARL2BP, a protein required for ciliary microtubule structure, cause syndromic male infertility in humans and mice. PLoS Genet. 15, e1008315. 10.1371/journal.pgen.1008315 31425546 PMC6715254

[B31] Nag P. Kumaresan A. Akshaya S. Manimaran A. Rajendran D. Paul N. (2021). Sperm phenotypic characteristics and oviduct binding ability are altered in breeding bulls with high sperm DNA fragmentation index. Theriogenology 172, 80–87. 10.1016/j.theriogenology.2021.06.006 34146972

[B32] Ni Z. Olsen J. B. Guo X. Zhong G. Ruan E. D. Marcon E. (2011). Control of the RNA polymerase II phosphorylation state in promoter regions by CTD interaction domain-containing proteins RPRD1A and RPRD1B. Transcription 2, 237–242. 10.4161/trns.2.5.17803 22231121 PMC3265782

[B33] Ostermeier G. C. Miller D. Huntriss J. D. Diamond M. P. Krawetz S. A. (2004). Reproductive biology: Delivering spermatozoan RNA to the oocyte. Nature 429, 154. 10.1038/429154a 15141202

[B34] Parthipan S. Selvaraju S. Somashekar L. Kolte A. P. Arangasamy A. Ravindra J. P. (2015). Spermatozoa input concentrations and RNA isolation methods on RNA yield and quality in bull (*Bos taurus*). Anal. Biochem. 482, 32–39. 10.1016/j.ab.2015.03.022 25823682

[B35] Paul N. Kumaresan A. Das Gupta M. Nag P. Guvvala P. R. Kuntareddi C. (2020). Transcriptomic profiling of buffalo spermatozoa reveals dysregulation of functionally relevant mRNAs in low-fertile bulls. Front. Vet. Sci. 7, 609518. 10.3389/fvets.2020.609518 33506000 PMC7829312

[B36] Prakash M. A. Kumaresan A. Ebenezer Samuel King J. P. Nag P. Sharma A. Sinha M. K. (2021). Comparative transcriptomic analysis of spermatozoa from high- and low-fertile crossbred bulls: Implications for fertility prediction. Front. Cell Dev. Biol. 9, 647717. 10.3389/fcell.2021.647717 34041237 PMC8141864

[B37] Rather H. A. Kumaresan A. Nag P. Kumar V. Nayak S. Batra V. (2020). Spermatozoa produced during winter are superior in terms of phenotypic characteristics and oviduct explants binding ability in the water buffalo (Bubalus bubalis). Reprod. Domest. Anim. 55, 1629–1637. 10.1111/rda.13824 32945545

[B38] Riesco M. F. Robles V. (2013). Cryopreservation causes genetic and epigenetic changes in zebrafish genital ridges. PLoS ONE 8, e67614. 10.1371/journal.pone.0067614 23805321 PMC3689738

[B39] Said T. M. Land J. A. (2011). Effects of advanced selection methods on sperm quality and ART outcome: A systematic review. Hum. Reprod. Update 17, 719–733. 10.1093/humupd/dmr032 21873262

[B40] Salvolini E. Buldreghini E. Lucarini G. Vignini A. Lenzi A. Di Primio R. (2013). Involvement of sperm plasma membrane and cytoskeletal proteins in human male infertility. Fertil. Steril. 99, 697–704. 10.1016/j.fertnstert.2012.10.042 23174138

[B41] Saraf K. K. Kumaresan A. Sinha M. K. Datta T. K. (2021). Spermatozoal transcripts associated with oxidative stress and mitochondrial membrane potential differ between high- and low-fertile crossbred bulls. Andrologia 53, e14029. 10.1111/and.14029 33665828

[B42] Saraf K. K. Singh R. K. Kumaresan A. Nayak S. Chhillar S. Lathika S. (2019). Sperm functional attributes and oviduct explant binding capacity differs between bulls with different fertility ratings in the water buffalo (Bubalus bubalis). Reprod. Fertil. Dev. 31, 395–403. 10.1071/RD17452 30135005

[B43] Singh R. K. Kumaresan A. Chhillar S. Rajak S. K. Tripathi U. K. Nayak S. (2016). Identification of suitable combinations of *in vitro* sperm-function test for the prediction of fertility in buffalo bull. Theriogenology 86, 2263–2271. 10.1016/j.theriogenology.2016.07.022 27555524

[B44] Valcarce D. G. Cartón-García F. Herráez M. P. Robles V. (2013). Effect of cryopreservation on human sperm messenger RNAs crucial for fertilization and early embryo development. Cryobiology 67, 84–90. 10.1016/j.cryobiol.2013.05.007 23727067

[B45] Vignesh K. Murugavel K. Antoine D. Prakash M. A. Saraf K. K. Nag P. (2020). The proportion of tyrosine phosphorylated spermatozoa in cryopreserved semen is negatively related to crossbred bull fertility. Theriogenology 149, 46–54. 10.1016/j.theriogenology.2020.03.020 32234650

[B46] Vijayalakshmy K. Kumar D. Virmani M. Jacob N. Kumar P. (2018). Sperm transcriptomics: An emerging technique to assess male fertility. Int. J. Curr. Microbiol. Appl. Sci. 7, 1188–1200. 10.20546/ijcmas.2018.709.141

[B47] Watson P. F. (1995). Recent developments and concepts in the cryopreservation of spermatozoa and the assessment of their post-thawing function. Reprod. Fertil. Dev. 7, 871–891. 10.1071/rd9950871 8711221

[B48] Watson P. F. (2000). The causes of reduced fertility with cryopreserved semen. Anim. Reprod. Sci. 60–61, 481–492. 10.1016/s0378-4320(00)00099-3 10844218

[B49] Wishart M. J. Dixon J. E. (2002). The archetype STYX/dead-phosphatase complexes with a spermatid mRNA-binding protein and is essential for normal sperm production. Proc. Natl. Acad. Sci. U. S. A. 99, 2112–2117. 10.1073/pnas.251686198 11842224 PMC122327

[B50] Wu K. Mei C. Chen Y. Guo L. Yu Y. Huang D. (2019). C-type natriuretic peptide regulates sperm capacitation by the cGMP/PKG signalling pathway via Ca2+ influx and tyrosine phosphorylation. Reprod. Biomed. Online 38, 289–299. 10.1016/j.rbmo.2018.11.025 30655075

[B51] Xia H. Chen Y. Wu K-J. Zhao H. Xiong C-L. Huang D-H. (2016). Role of C-type natriuretic peptide in the function of normal human sperm. Asian J. Androl. 18, 80–84. 10.4103/1008-682X.150254 25926602 PMC4736361

[B52] Yeste M. (2016). Sperm cryopreservation update: Cryodamage, markers, and factors affecting the sperm freezability in pigs. Theriogenology 85, 47–64. 10.1016/j.theriogenology.2015.09.047 26506124

[B53] Zhang K. Wang H. Rajput S. K. Folger J. K. Smith G. W. (2018). Characterization of H3.3 and HIRA expression and function in bovine early embryos. Mol. Reprod. Dev. 85, 106–116. 10.1002/mrd.22939 29232016

