# Supplementary figures and images for: Cryopreservation process alters the expression of genes involved in pathways associated with the fertility of bull spermatozoa
Source: Front Genet. 2022 Oct 25;13:1025004. doi: 10.3389/fgene.2022.1025004 (PMC9640914; doi:10.3389/fgene.2022.1025004)

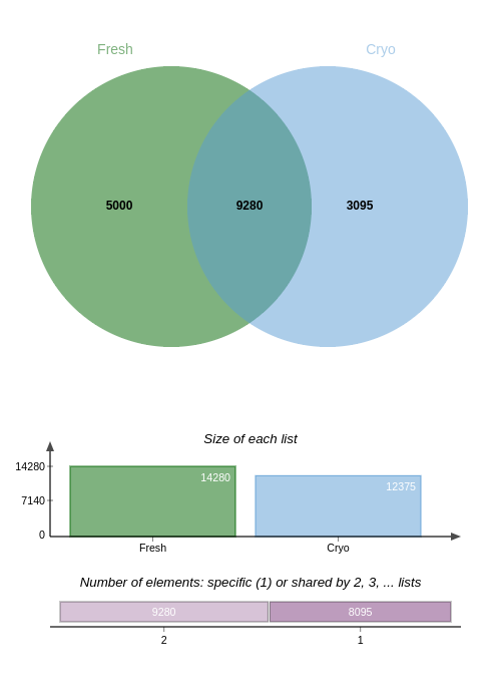

Supplement: Supplementary file 4 [file Image1.TIF]
